# The Medical Society of the County of New York

**Published:** 1879-08

**Authors:** 


### Reports of Societies.

### THE MEDICAL SOCIETY OF THE COUNTY OF NEW YORK.

*Treatment of Infantile Diarrhœa.*—Dr. A. Jacobi read an important paper on the above subject, of which the following is an abstract:

Of all the deaths in the first year of life forty per cent. in round numbers were due to diseases of the digestive organs, and half as many to such of the respiratory organs. In the second year the main cause of death changed completely; for of all the forty-five per cent. of deaths taking place in that year, but nine were due to digestive, and thirty-six per cent. to respiratory disorders. Thus in the first year, stomach and intestines; in the second, bronchi and lungs, were the sources of high death-rates.

Mortality diminished with every day of advancing life.

Every additional hour improved the baby's chances for preservation. Almost one-half of the infants dead before the end of the first year died before they were one month old. Thus the causes of disease were the more active the earlier they were brought to bear upon the young with their defective vitality.

Two grave conclusions were to be drawn from that fact. The first was, that the diminution of early mortality depended on avoiding diseases of the digestive organs by insisting upon normal alimentation. That was principally important in the first few months. While breast milk had been shown to lower infant mortality through the whole first year, it did so more in the few first months. Thus, though an infant might not be fed on breast-milk through the whole normal period of nursing, a great gain, indeed, was accomplished by insisting on nursing, though for a limited time, perhaps two months only. There were but few mothers but were capable of nursing during that brief time, and none who ought to be spared the accusation of causing ill health or death to her baby if she refused to nurse it at least through the first dangerous months. The second conclusion, resulting from many figures, was that the dietetic problems and rules for the infant concerned the digestive organs mainly, so much so indeed, that infant dietetics and the dietetics of the infant digestive organs appeared nearly identical.

It was true that in this city we met with a high mortality, even in children of more than a year. In fact, public opinion looked for a higher mortality in the second than in the first summer. The fallacy of that assumption could be easily corrected by the statistical reports. The second summer was the period of danger in part only because of the heat of the season, but mainly of the errors in feeding. Conscientious and intelligent families in good circumstances were not apt to lose their infants in their second summer.

Nor was it necessary that he should insist upon the danger incurred by the belief that diarrhœa—a pathological condition—was a normal attendant on, and a relief of, a physiological process, such as dentition. It was certain that very few, if any, popular beliefs had been more destructive than that an intestinal catarrh must be left alone, no matter from what source it originated.

Healthy infants had a normal tendency to loose, liquid or semi-fluid evacuations from the bowels. The causes were partly in the condition of the intestinal tract, and partly in the nature of the normal food, viz: breast-milk. The peristaltic movements were very active; the young blood-vessels were very permeable; the transformations of surface cells very rapid; the peripheric nerves very superficial, more so than in the adult, whose mucous membrane and submucous tissue had undergone thickening by both normal development and morbid processes. In the young infant the peripheric ends of the nerves were larger in proportion than in the adult; the anterior horns of the nerve-centres were more developed than the posterior ones. Thus the greater reflex irritabilty of the young, particularly in regard to intestinal influences, was easily explained. Besides, the action of the sphincter ani was not quite powerful, the fæces were not retained in the colon and rectum, and no time was afforded for the reabsorption of the liquid or dissolved constituents of the fæces. Moreover, the frequency of acids, sometimes normal, in the small intestines gave rise to the formation of alkaline salts with purgative properties.

The nature of breast-milk, even when absolutely normal, was such as to facilitate frequent, large, and fluid evacuations.

*Fat.*—First, as to its fat where no food was given but mother's milk, *a good deal of fat was eliminated* without any change.

What had been called detritus in the fæces was mostly fat, and very probably remnants of intestinal epithelium. Practically that *fact* was of the very greatest importance. The conclusion was, we were almost certain to give too much fat; it was scarcely ever probable that there was too little. Therefore, the addition of cream was reprehensible, no matter in what shape. Thus in the most normal milk there was more fat than required.

In the first period of lactation the glandular transformation was not yet accomplished. It required days to exhibit casein. At the same time the percentage of butter and salts was very high indeed, both of which explained the laxative character of colostrum.

There was no stability in the nature of breast-milk, and very much less in the human than in the animal female, for obvious reasons. Its constituents and effects might even change from hour to hour, from day to day, sometimes it would be milk, sometimes milk with transuded serum.

*Sugar.*—Again, as to sugar. It was abnormally plentiful in colostrum, and in some milk, at times, its percentage was lower than normal. In the former it was purgative, in the latter its absence one of the causes of constipation. Thus the addition of a piece of sugar—which need not be milk sugar—to breast-milk was apt to heal constipation in the infant. He dissolved it in the smallest possible quantity of water, say a teaspoonful, and let the baby take it before each nursing.

*Casein.*—Fourthly, as to casein. When present in an abnormally high percentage, it might either constipate or by remaining undigested, and, acting as a local irritant, produce a diarrhœa. In those cases of diarrhœa the stools were mixed with white flocculi, small or large, sometimes in astonishing quantities and for a long period. The treatment of such diarrhœa was by no means very simple, unless the breast-milk was changed. When such a change could not take place, he added oatmeal gruel or barley-water in such a manner that a few teaspoonfuls of it were administered to the baby before each nursing.

Dr. Jacobi then passed to the examination of some of the articles of food mostly used for the young.

Goat's milk ought to be rejected because of its lage percentage of fat.

Cow's milk contained more butter than human milk, and if the latter was not entirely digested, the former would certainly leave even more remnants to encumber the intestinal canal. The reaction of human milk was alkaline, that of cow's milk rarely to the same degree.

But the main difficulty was in the large percentage and in the nature of the casein of cow's milk. The casein of cow's milk and the casein of woman's milk were two different substances. The former had an acid relation and was soluble in water in the portion of 1–20. The latter was alkaline or neutral, and almost entirely soluble in water. There was less casein in women's milk than in cow's milk. The fact was beyond doubt that pure cow's casein was very much less digestible than human casein.

At all events, no addition we knew of could render cow's casein more digestible than Nature made it, and the only thing which could be obtained by any sort of manipulation of the milk was to make it less injurious. Perhaps, however, the plan upon which Dr. J. Rudisch had acted might recommend itself to the attention of the practitioner. In order to make cow's milk more digestible, he had introduced into Dr. Jacobi's practice a mixture which promised to be of great value in all those cases in which coagulability of the mik was the prominent obstacle to its usefulness. The mixture suggested by him, and used by them up to that time mainly in diseases of adults, such as anæmia, gastric catarrh, ulcer of the stumach, slow convalescence, etc., was the following: to one pint of water add one-half teaspoonful of officinal dilute muriatic acid. To that mixture add one quart of cold cow milk; mix the two liquids thoroughly and then boil for ten or fifteen minutes. He had found that preparation to be very digestible, and well tolerated by very feeble digestive organs. Valuable as that preparation of cow's milk might prove in future, there was one method for making cow's milk more available, which was at once simple and effective. No cow's milk ought to be administered without the addition of choride of sodium. Not only cow's milk, but also —and even much more so—farinaceous admixtures to cow's milk required its presence in the food.

*Preventive Treatment.*—The preventive treatment of diarrhœa, depending on defective alimentation, consisted in so changing and arranging the milk used for babies that the casein would not coagulate in large lumps, and thus become more digestible. That object could be obtained by adding such farinaceous food as did not contain much starch. It consists in diluting the boiled and skimmed milk with barley-water or oatmeal gruel. It must be boiled to check its tendency to become sour, to remove a portion, though small, of its casein and fat, and to expel the gas contained in the raw milk to the amount of three per cent.

Of the two, he preferred barley for general use. He recommended that the barleycorn which was employed for infant diet should be ground as thoroughly as possible in a coffee-mill, both in order to diminish the period necessary for cooking it, and also in order to retain the gluten. *It was even preferable, for very young infants, to cook the barley whole for hours*, thereby to burst the outer layers of cells, empty their contents, and then, by straining, to get rid of the larger part of the starch which was found toward the center. There was no danger to which little children were so liable as that which arose from their tendency to diarrhœa. His advice, therefore, was to administer barley to children who manifested a tendency to diarrhœa, and oatmeal to those having a tendency to constipation, and, whenever a change occurred in the intestinal functions, to give one or the other, according as constipation or diarrhœa predominated.

He held that the mixture to be the *conditio sine qua non* of the thorough digestion of the milk. It only would insure the proper nourishment of the infant. With that food alone he had seen children endure the heat of summer without any attack of illness whatever. He had occasion again and again to be convinced of the reliability of the mixture. It had the advantage, too, that it necessitated no dependence upon the honesty or competence of the apothecary or manufacturer, but could be prepared by any one, however poorly situated. Should a slight diarrhœa occur, or a little casein be vomited (a rare accident, to be sure,) or casein occur in the stools, then all that was necessary was to diminish the proportion of milk. It might sometimes be necessary, though very seldom, to withdraw the milk entirely for a time, but only in cases of real illness. If the physician or attendants had properly apportioned the ingredients of the mixture, we might be rather sure that the child's digestion and assimilation would be regular and normal. Infants that were partly nourished at the breast almost invariably thrived well with the addition of his mixture· Children from their fourth or fifth month and upward, might often be fed with it exclusively, and not unfrequently nothing else was given from the day of the birth.

The addition of barley or oatmeal for the purpose of rendering milk digestible was not, however, absolutely indispensable, though he had learned to prefer them. Gum-arabic and gelatine were also very valuable ingredients, indeed, of infant foods. Dr. Jacobi then dwelt at some length upon the changes which gum-arabic and gelatine undergo when put into the stomach.

*Curative Treatment.*—The amount of food should not be larger than we had reason to expect could be easily digested. At all events, either lengthen the intervals between the meals or reduce the quantity of food given at one time, or both. When diarrhœa made its appearance in infants who had been weaned, it was desirable to return them to the breast. Those who never had breast milk might be given the breast if they could be induced to take it, but only rarely would that be found possible. Whenever a child at the breast was taken with diarrhœa, the passages from the bowels should be studied as to their contents. If a certain amount of curd was found in them, the least that was to be done was to mix the breast-milk with barley-water. That might be done in such a manner that, each time before nursing, one or two teaspoonfuls of barley-water was given the child, so that the farinaceous food and the breast-milk mixed in the stomach. Or, it might be found advisable to alternate breast-milk and barley-water. In bad cases, particularly when the milk was found to be white and heavy, and contained a great deal of casein, it would be found necessary to deprive the child *altogether* of its usual food. In such cases, the child would do better on barley-water alone (that to be continucd for one or two days), than to expose it to the injury which would certainly follow the continuation of the casein food.

When diarrhœa occurred in children who had been fed alone upon cow's milk, unmixed or mixed, it was necessary to reduce the quantity of cow's milk in the mixture. As a rule, we had to remember that cow's milk alone was apt to produce diarrhœa, and it should be considered as a maxim that, whenever diarrhœa made its appearance, the amount of cow's milk given to the child should be reduced. When a mere reduction of the quantity did not suffice, it was very much better to deprive the child of milk food altogether. Not infrequently the removal of milk from the bill of fare was the only thing which would restore the child to health. It was possible that a mixture, such as recommended by Dr. Rudish, already mentioned, would be found digestible, even in such cases. In many cases, as a dietetic measure, it would be found advisable to add one or two tablespoonfuls of lime-water to each bottle of food with which the child was supplied.

In those cases in which barley-water did not seem to suffice as a nutriment, or where it would be dangerous to allow children to lose strength, a mixture which he had used to great advantage was the following: Mix the white of one egg with four or six ounces of barley-water, and add a small quantity of table salt and sugar, just sufficient to make the mixture palatable. The child could take this either in large or small quantities, according to the case.

In those cases in which the stomach was irritable, and vomiting had occurred, it was now and then better to give a small quantity, even one or two teaspoonfuls, and repeat the dose every ten, fifteen or twenty minutes, than to give larger quantities at longer intervals.

In those cases in which the strength of the child has suffered greatly, he recommended the addition of brandy to the mixture in such quantity that the child would take from one drachm to one ounce (grms. 1.0 to 30.0,) more or less, in the course of twenty-four hours.

In those extreme cases in which the intestinal catarrh was complicated with gastric catarrh, where the passages were numerous and copious, and vomiting constant, where both medicines and food were rejected, there was frequently but one way to save the patients, and that was to deprive them *absolutely* of everything in the form of either drink or food or medicine. It was true that such babies would suffer greatly from thirst for an hour or two, but it was a fact that, after two or three hours, those children would look better than before the abstemious treatment was commenced. Not unfrequently four or five hours of total abstinence would suffice to quiet the stomach and diminish both the secretion and the peristaltic movement of the intestinal tract. In some cases *six* or *eight* hours of complete abstinence would be required; or such children might be starved for even *twelve* or *sixteen* hours, with final good results The first meals afterward must be quite small, and they would be retained, and, as a rule, such children would subsequently do well.

Dr. Jacobi here enforced the necessity of supplying the patient with as much cool fresh air as possible. The worst out-door air was better than close in-door air. If possible, the children should be sent immediately to the country and into the mountain air.

*The second indication* consisted in the removal of undigested masses retained in the intestinal tract. Not only in cases in which the diarrhœa had resulted from previous errors in diet of the child, but also in those cases dependent upon sudden changes of temperature and exposure, it was desirable to empty the intestinal tract. For that purpose castor-oil, calcined magnesia, or calomel might be used.

*Third.* Nothing should be given that contained salts in any sort of concentration. Thus, beef-tea should be avoided. It must be remembered that that form of meat-extract contained a very large amount of salts, and that the direct effect of these upon the intestinal canal might be productive of very unpleasant consequences. If the people insisted upon giving it, and there was no special contra-indication to its use in a given case, it should be administered only in connection with some well-cooked farinaceous vehicle, and the best of all for that purpose was barley-water; or it might be mixed with beaten white of egg, but no more chloride sodium should be added. For the main danger in beef-tea was the concentrated form in which its salts were given.

*Fourth.* Everything should be avoided that increased peristaltic motion. Thus, carbonic acid and ice internally.

*Fifth.* Avoid whatever threatened to increase the amount of acid in the stomach and intestinal tract. There was so much acid in the normal, and still more in the abnormal stomach and intestinal tract, that it was absolutely necessary to *neutralize* it. For that purpose it was safer to resort to preparation of calcium than of sodium or magnesium. So far as lime-water was concerned, its administration, certainly, was correct chemically. But we should not place too much reliance upon that popular remedy. We should not forget that it contained about one part of lime to eight hundred of water, and that it was necessary to swallow at least two ounces of the fluid in order to obtain a single grain of lime.

A further indication was, the necessity of destroying ferments. For that purpose most metallic preparations would do fair service. One which had been extensively used was calomel, and now in small doses frequently repeated—1 10, $\frac{1}{4}$, or $\frac{1}{2}$ a grain (0.1 0.15 0.03) every two hours. As to its effect as an anti-fermentative, there could be no doubt.

*Nitrate of Silver*, when given for the same purpose, should be largely diluted. From 1-40 to 1-16 of a grain (0.0015 0.004) dissolved in a teaspoonful or tablespoonful of water, might be given every two or three hours, and not infrequently with fair result. That was especially important with regard to injections of nitrate of silver into the rectum, where it was apt to do as much harm as good. Whenever it was to be given in that way, the anus and its neighborhood should be washed with salt water before the injection was administered.

*Bismuth* acted very favorably. Moderate cases of diarrhœa would usually show its effect very soon. Doses of from $\frac{1}{2}$ to 2 or 3 grains (0.03 0.20) given every two or three hours, would act very favorably indeed. In those cases in which the diarrhœa had lasted for a long time, the doses of bismuth should be large in order to be certain of immediate contact of the drug with the sore surface.

A final indication was the depression of the hyperæsthesia of the general system and of the intestinal tract in particular. There had been authors who condemned the use of opium altogether, which, certainly, was incorrect. The doses should be small, and they might be frequently repeated. Administered in that manner, opium could be used with perfect safety both internally and in an enema. One of the rules for giving opium was that the child should not be waked up for the purpose of taking the medicine. Whenever there was fear of collapse, it was safer to give 1-200 of a grain (0.0003) every half hour or hour, than to administer 1-50 of a grain (0 0012) every two hours.

*Alcohol.*—Small and frequent doses would certainly stimulate the nervous system, digestion, and circulation, and they also stimulated the skin and increased perspiration. Alcohol, given in that manner, certainly arrested fermentation. Moreover, it took the place of food, and acted favorably as food when no solid carbo-hydrates were tolerated by the intestinal tract. As it was absorbed in the stomach, so did it protect the intestinal tract.

*Finally*, it is necessary to reduce the amount of secretion taking place from the surface of the intestinal tract. For that purpose astringents might be used, such as alum, lead, tannic acid, pernitrate of iron, and, what had already been spoken of, nitrate of silver. In all those cases in which the stomach participated in the process to any considerable extent, almost any astringent would prove ineffective. To fulfil several indications at the same time, it was often good practice to combine remedies.

The main indications were to neutralize acids, to reduce nervous irritability, to arrest secretion, and to change the condition of the surface of the catarrhal mucous membrane.

For that purpose, in the generality of cases, he combined bismuth, opium, and chalk, according to the following formula:

R. Bismuth subnit.....................gr. i. (0.05)
Prepared chalk.....................grs. ij. (0 10–0.20)
Dover's powder.....................gr. $\frac{1}{3}$ (0.02)

That combination was suitable for a baby ten or twelve months of age, and the dose could be repeated every two hours. In all those cases in which acid was very abundant, it was necessary to increase the doses of antacids without necessarily giving large doses of opium.

Hot bathing was especially serviceable in those cases in which the surface was cool and the temperature of the body, measured in the rectum, was pretty high. To relieve intestinal pain, plain warm fermentations; to relieve heat, cold applications were sufficient.

*Camphor* stimulated the heart, and reduced temperature, and might be used internally or subcutaneously, according to the necessities in the case. For subcutaneous injections it might be dissolved in either oil or alcohol. The effect derived from camphor as a stimulant was not permanent, but very much more so than that produced by carbonate of ammonia. The dose might be from $\frac{1}{4}$ to $\frac{1}{2}$ a grain (0.15 0.03) every hour or two, when only a moderate stimulation was required. In urgent cases it might be given in doses of from five to ten grains in the course of an hour, and usually the effect would be favorable. It was, however, only in cases in which real collapse was present that doses of five or ten grains would be required.

There was no remedy that would act more favorably in conditions of great debility and collapse than musk. It might be given in doses of five or ten grains, and repeated every half hour or hour. More than two or three such doses would not be required to yield a result.

The paper being before the Society for discussion,

Dr. Wm. H. Thomson remarked that, doubtless, each one of us had, by experience and reflection upon that experience, been led to the conclusion that in the course of years we became more or less a law to ourselves. He was very glad that the opportunity had been afforded for some one to come in contact with his own ideas, and none more acceptably than the author of the paper, who was one of the most eminent authorities in his department in this county. In certain respects he disagreed with Dr. Jacobi, and was sure that he dreaded the beginning of dentition, as having a direct bearing upon the etiology of some of the more serious forms of summer diarrhœa, which occasionally occurred with the suddenness and explosiveness of true cholera. The reflex effect from the irritation incident to dentition was to suspend the secretion of gastric juice, hence he had been led to administer bromide of potassium early in the treatment of infantile diarrhœa, and, as he thought, with good results. He employed it as an agent that arrested reflex irritation. He had been led to believe there was a difference between the diarrhœa due to local irritation, such as teething or fermentation of the contents of the intestines, and the diarrhœa which seemed to be due to sudden nervous paralysis. The one he would regard as a catarrhal diarrhœa, and the other as a true choleraic diarrhœa. It had appeared to him that there was a difference between the two classes of cases from the very beginning. The manner in which the choleraic diarrhœa began—without any evidence of distress accompanying or preceding the discharges, but the existence of large watery passages and their sudden development of grave cerebral symptoms—had led him to regard its access as very often due to an infection of a kind not dissimilar to Asiatic cholera. It was in the choleraic class of cases in which the effect of camphor seemed to be beneficial, and to be indicated from the very commencement. He always ordered camphor or some remedy allied to camphor in any case of diarrhœa in which there is much looseness of the bowels without pain, and he directed special attention to that point, to be noticed by the attendants upon children in warm weather. Camphor, unquestionably, was a substance which was not dissimilar to carbolic acid and the whole array of spices, which were antiseptics when taken internally. He relied very much upon them. The action of spices was very similar to the action of camphor, and in some cases peppermint and allspice were combined with small doses of camphor, preferably in those cases in which there was great irritability of the stomach.

Lastly, as to the question of the use of hot water for the purpose of restoring the circulation in cases of threatened collapse, he fully agreed with Dr. Jacobi in the employment of the agent for that purpose, for he had used it with great success to overcome sudden and exceedingly dangerous contraction of the peripheral arteries. He used large hot-water injections, and ordered them to be given as freely as possible. He thought the hot water acted in two ways: 1. It aroused the patient; and 2. It allayed the peristaltic action of the intestines. For dysentery he gave but little medicine, but constantly washed out the lower bowel with hot-water injections. Finding that it was equally well borne in the dysentery of children as adults, and allayed the irritability of chronic dysentery, he tried it also in cholera infantum, and strongly recommended its use.

The Society then adjourned.

